# Characterization of the Proprotein Convertase-Mediated Processing of Peroxidasin and Peroxidasin-like Protein

**DOI:** 10.3390/antiox10101565

**Published:** 2021-09-30

**Authors:** Hajnal A. Kovács, Enikő Lázár, György Várady, Gábor Sirokmány, Miklós Geiszt

**Affiliations:** 1 Department of Physiology, Faculty of Medicine, Semmelweis University, 1085 Budapest, Hungary; kovacs.hajnal@med.semmelweis-univ.hu (H.A.K.); lazar.encsi@gmail.com (E.L.); sirokmany.gabor@med.semmelweis-univ.hu (G.S.); 2 Peroxidase Enzyme Research Group, Semmelweis University, 1085 Budapest, Hungary; 3 Research Centre for Natural Sciences, Institute of Enzymology, 1117 Budapest, Hungary; varady.gyorgy@ttk.mta.hu

**Keywords:** hydrogen peroxide, peroxidase, reactive oxygen species, extracellular matrix, basement membrane, peroxidasin, peroxidasin-like protein, collagen IV, sulfilimine bond

## Abstract

Peroxidasin (PXDN) and peroxidasin-like protein (PXDNL) are members of the peroxidase-cyclooxygenase superfamily. PXDN functions in basement membrane synthesis by forming collagen IV crosslinks, while the function of PXDNL remains practically unknown. In this work, we characterized the post-translational proteolytic processing of PXDN and PXDNL. Using a novel knock-in mouse model, we demonstrate that the proteolytic cleavage of PXDN occurs in vivo. With the help of furin-specific siRNA we also demonstrate that the proprotein-convertase, furin participates in the proteolytic processing of PXDN. Furthermore, we demonstrate that only the proteolytically processed PXDN integrates into the extracellular matrix, highlighting the importance of the proteolysis step in PXDN’s collagen IV-crosslinking activity. We also provide multiple lines of evidence for the importance of peroxidase activity in the proteolytic processing of PXDN. Finally, we show that PXDNL does not undergo proteolytic processing, despite containing sequence elements efficiently recognized by proprotein convertases. Collectively, our observations suggest a previously unknown protein quality control during PXDN synthesis and the importance of the peroxidase activity of PXDN in this process.

## 1. Introduction

Reactive oxygen species (ROS) have divergent physiological functions in living organisms, and their role in disease formation is also increasingly recognized. Some of the biological effects of ROS are mediated through enzymes that belong to the peroxidase-cyclooxygenase superfamily [1,2]. Six members of this enzyme family are present in mammals: myeloperoxidase (MPO), eosinophil peroxidase (EPO), lactoperoxidase (LPO), thyroperoxidase (TPO), peroxidasin (PXDN), and peroxidasin-like protein (PXDNL) (also known as HsPxd02 and VPO2) [1,2,3]. While the biological role of PXDNL is currently unclear, the other members of the family function in host defense (MPO, EPO, LPO), hormone synthesis (TPO), while PXDN participates in the formation of the extracellular matrix (ECM). PXDN was originally identified in Drosophila melanogaster [4], but subsequent studies proved that this enzyme is expressed in several other species, including mammals [5,6]. What is unique about PXDN protein structure is that it contains protein modules along with the peroxidase domain that are characteristic of the components of the extracellular matrix. These protein modules include leucine-rich repeats and immunoglobulin-like domains, which are located *N*-terminally to the peroxidase domain and a C-terminal von Willebrand factor (vWF)-like domain [4]. The presence of these protein domains suggested that the enzyme had a role in building the ECM as it was proposed in Drosophila [4] and demonstrated in differentiating mammalian fibroblasts [6]. The biochemical function of PXDN was first discovered by Bhave et al., who demonstrated that PXDN catalyzes the formation of sulfilimine crosslinks between the NC1 domains of collagen IV protomers during the synthesis of basement membranes [7]. In this reaction, bromide ions are oxidized by PXDN into hypobromous acid, and this compound oxidizes highly conserved amino acid residues to form the crosslinks [8]. Although there are many unknowns regarding the exact mechanism and significance of the PXDN-catalyzed reaction, our knowledge of PXDN is rapidly expanding. Two groups, including ours, have demonstrated that the mammalian enzyme functions in trimers as it was initially suggested for the Drosophila homolog [9,10]. It was also recently described that the enzyme mediates the bromination of tyrosine residues but the physiological significance of this activity remains to be clarified [11,12].

Our knowledge on PXDNL is more limited. The protein shows a high degree of homology to PXDN, but highly conserved amino acids are replaced by other residues in its peroxidase domain [13]. These critical changes explain the lack of peroxidase activity of heterologously-expressed PXDNL [13]. The PXDNL gene is present in the human genome, but is absent in the mouse and rat genomes. The PXDNL protein is expressed in the human heart, where it localizes to cardiomyocytes [13].

While analyzing the structural features of PXDN, Colon et al. identified a proprotein-convertase cleavage site at the C-terminus of the protein and demonstrated PXDN cleavage in transfected cells [14]. According to their analysis, the location of the cleavage is at Arg1336.

The current study aimed to explore whether this cleavage also occurs in vivo in animal tissues, and we also wanted to characterize this maturation step with a special emphasis on its structural requirements and impact on PXDN function.

Using a novel knock-in mouse model, we demonstrate that the cleavage of PXDN also occurs in vivo. With the help of genetically-modified cell culture model, we also show that only the proteolytically processed PXDN integrates into the extracellular matrix, explaining the importance of the proteolysis step in collagen IV-crosslinking. We also provide several lines of evidence for the importance of peroxidase activity in the proteolytic processing of PXDN. Notably, we also show that PXDNL is not cleaved, despite containing sequence elements recognized by proprotein convertases. Collectively, our observations suggest a previously unrecognized protein quality control mechanism in PXDN synthesis.

## 2. Materials and Methods

### 2.1. Primary Antibodies

HA-tag (C29F4) rabbit monoclonal antibody was obtained from Cell Signaling Technology (Danvers, MA, USA), and we used it in Western blot analysis. V5-tag mouse monoclonal antibody was purchased from AbD Serotec (Raleigh, NC, USA), and we used it in Western blot experiments, and we used an anti-V5 Epitope tag rabbit polyclonal antibody (Sigma-Aldrich, St. Louis, MO, USA, AB3792) in immunostaining experiments. Monoclonal Anti-FLAG M2 antibody produced in mouse (Sigma-Aldrich, F3165-1MG) was applied in both Western blot and immunostaining procedures. Anti-β-actin or anti-vinculin mouse monoclonal antibody (Sigma-Aldrich) was used to detect loading controls in the Western blot experiments. Anti-Human α1(IV) NC1 clone H11 (Chondrex, Woodinville, WA, USA) was also used in Western blot analysis. A mouse, anti-PXDN polyclonal antibody was raised by immunizing mice with a GST-fusion protein containing a 181 amino acids long fragment (aa 428-609) of the mouse PXDN protein.

### 2.2. Generation of Recombinant PXDN Constructs

Open reading frame of the human PXDN cDNA (NM_012293.3) was inserted into pcDNA3.1/V5-His-TOPO vector with TA cloning strategy (Invitrogen, Life Technologies, Waltham, MA, USA). Using site-directed mutagenesis, a silent mutation was introduced directly after the nucleotide sequence corresponding to the secretory signal (‘s’, aa1-26), thus creating a unique NheI restriction site. In a next step, hybridized oligonucleotides (Sigma-Aldrich) coding for a 3 × FLAG tag were inserted into this position with their overhangs matching those of the NheI-digested plasmid, resulting in a full-length wild-type PXDN construct with an *N*-terminal 3 × FLAG and a C-terminal V5-6 × His label (s-3 × FLAG-WT-V5). We used this construct as a starting point to clone several mutant forms of the human PXDN protein.

To limit the localization of the recombinant PXDN to the intracellular space, we modified the wild-type PXDN with a C-terminal endoplasmic reticulum retention signal, thus creating the s-3 × FLAG-WT-V5-KDEL (KDEL) construct. For this purpose, an oligonucleotide region corresponding to the KDEL amino acid sequence (Sigma-Aldrich) was added directly to the 3′-end of the V5-coding region, using its ends complementary to the ones created by AgeI cleavage (Fermentas, Waltham, MA, USA) in pcDNA3.1/V5-His-TOPO plasmid’s multiple cloning site.

The insertion and the correct orientation of the oligonucleotide regions were checked with restriction digestion (Fermentas) and further confirmed by sequencing (Eurofins MWG Operon, Ebersberg, Germany).

Originating from the wild type PXDN construct, we have also generated several point mutants (abbreviations in parenthesis are used in this paper): s-3 × FLAG-RR/AA1335-1336-V5 (RR/AA1335-1336); s-3 × FLAG-RK/AA1354-1355-V5 (RK/AA1354-1355); s-3 × FLAG-RR/AA1335-1336, RK/AA1354-1355-V5 (double mutant); s-3 × FLAG-C/S736, 1315-V5 (C736S, C1315S); s-3 × FLAG-RGR/QKK 1333-1335-V5 (RGR/QKK1333-1335); s-3 × FLAG-Q/W 823 D/E 826-V5 (Q823W, D826E)) with a double-primer PCR method using complementary mutagenic oligonucleotide pairs (Sigma-Aldrich). Mutated constructs were amplified with Phusion Hot Start II High-Fidelity DNA Polymerase (Thermo Scientific, Waltham, MA, USA). DNA regions containing the desired nucleotide changes were sequenced (Eurofins MWG Operon) and cloned back into the vector backbone using suitable restriction endonuclease pairs (Fermentas, Thermo Scientific). cDNA encoding full-length human PXDNL was inserted into pcDNA3.1/V5-His-TOPO vector with TA cloning strategy (Invitrogen, Life Technologies). An oligonucleotide encoding the first 995 nucleotides (from ATG) of the cDNA and including a 3 × FLAG tag coding region after the signal sequence was synthesized (BioCat GmbH, Heidelberg, Germany) and digested with HindIII (Fermentas) then ligated into the HindIII digested full length human PXDNL coding pcDNA3.1/V5-His-TOPO vector backbone. The final sequence was checked with sequencing (Microsynth AG, Balgach, Switzerland).

### 2.3. Generation of Transgenic Mice

To create knock-in mice expressing a hemagglutinin (HA)-tagged form of PXDN, a 2 × HA tag was inserted into the PXDN gene’s first exon/coding region after the 23rd amino acid, using CRISPR/Cas9 technology for genome editing [15]. We used Alt-R Cas9 protein (from IDT, Coralville, IA, USA) with cr-/tracrRNAs, with the CTTCGAGGCGACCACCGCCA target sequence (XM_006515205.4) on the crRNA. The 2 × HA tag sequence, and the homology arms, was synthesized as an ultramer single-stranded oligonucleotide (ssODN, IDT). Microinjection was carried out into the pronuclei of fertilized eggs of FVB/Ant mice. Concentrations of the injection solution were: 30 ng/µL Cas9 protein, 1 pmol/µL, ssODN 15 ng/µL in IDTE buffer. The generation of the PXDN knockout mouse was described in [10]. Animal experiments were approved by the National Scientific Ethical Committee on Animal Experimentation.

### 2.4. Mouse Embryonic Fibroblast (MEF) Preparation

Mouse fetuses were collected from HA-PXDN transgenic pregnant female mouse between day 12.5–14.5 of gestation. The embryo’s head, liver and blood clots were removed, then the remaining body was washed with sterile PBS and minced in a sterile dish into little pieces. The body pieces (from 5–6 embryos) were incubated in 2 mL Trypsin (Lonza, Basel, Switzerland) for 10min at 37 °C. After the incubation the digested suspension was vigorously pipetted up and down to make a single cell suspension. 1 mL of the suspension was placed in a 25 cm^2^ flask into 4 mL freshly prepared MEF medium. After passage 1 multiple flasks were frozen for long term storage purposes.

### 2.5. Cell Culture and Treatments

PFHR-9 cells (ATCC, Manassas, VA, USA, CRL-2423) and the PXDN-KO PFHR-9 cells were cultured in Dulbecco’s modified Eagle’s medium (DMEM) with glutamine and 4.5 g/L glucose, supplemented with 10% fetal bovine serum (Lonza) and 50 U/mL penicillin and 50 µg/mL streptomycin (Lonza). The protocol of the generation of the PXDN KO cell line was published before by our laboratory [16]. The HA-PXDN MEF cells were cultured in Dulbecco’s modified Eagle’s medium (DMEM) with glutamine and 4.5 g/L glucose, supplemented with 10% fetal bovine serum (Lonza), MEM non-essential amino acids (Gibco, Waltham, MA, USA, 50 U/mL penicillin and 50 µg/mL streptomycin (Lonza). The cells were grown in a humidified incubator at 37 °C, in 5% CO_2_ containing air. We used Lipofectamine LTX and Plus Reagents (Life Technologies) for transient transfections of PXDN-KO PFHR-9 cells. For immunostaining and Western blot experiments, the cells were transiently transfected with different pcDNA3.1/FLAG-PXDN-V5-His and with pcDNA3.1/FLAG-PXDNL-V5-His constructs by following the instructions of the manufacturer. Two days after the transfection, the cells were harvested for protein sample preparation. In the case of phloroglucinol treatment, the drug was added to the medium in 50 µM final concentration during the two days. For immunostaining, the cells were grown on glass coverslips for three days after their transfection, and their medium was supplemented with 50 µg/mL vitamin C. PFHR-9 cells were transfected with small interfering (si)RNA at 40 nM concentration using the Lipofectamine™ RNAiMAX Transfection Reagent. The siRNA was either Furin Silencer Select Pre-designed siRNA (Ambion, Austin, TX, USA, cat. no. 4390771) or as negative control we used Silencer Select Negative Control #1 siRNA (Ambion, cat. no. 4390843). During the proprotein convertase inhibitor experiment PFHR-9 cells were grown in cell culture for three days and the inhibitor’s solvent DMSO or the furin inhibitor Decanoyl-Arg-Val-Lys-Arg-Chloromethylketone (CMK) was added to the medium at 50 µM final concentration in the last 24 h. The HA-PXDN MEF cells were grown in cell culture for seven days and their medium was supplemented with 50 µg/mL vitamin C. In the last 24 h DMSO or CMK was added to the medium in the same concentration as in the case of the PFHR-9 cells.

### 2.6. Immunofluorescent Labeling and Confocal Laser Microscopy

Three days after their transfection with various PXDN constructs, the PXDN-KO PFHR-9 cells were fixed in cold acetone on ice for 5 min. After washing with PBS several times, the cells were incubated with 6 M urea and 100 mM glycine, pH 3.3 for 25 min, then washed 4 times again with PBS. Next, the samples were blocked with 5% BSA in PBS for 1 h at room temperature (RT). The primary antibody was added to the blocking solution in 1:500 dilution, and the samples were incubated overnight (ON) at 4 °C in a humidified glass chamber. On the next day, they were washed with PBS for 30 min then incubated with the secondary antibody (Alexa Fluor488 donkey anti-mouse IgG (H + L) and Alexa Fluor568 donkey anti-rabbit IgG (H + L) were purchased from Life Technologies) and with TO-PRO-3 iodide (642/661) (purchased from Life Technologies) for nuclear staining at RT for 1 h in darkness. Finally, the samples were washed again with PBS for 30 min. In the end, we used Mowiol 4-88 (Sigma Aldrich, St. Louis, MO, USA) antifade reagent for mounting.

Frozen mouse embryo’s head (dissected between day 12.5–14.5 of gestation) sections were fixed in cold acetone on ice for 5 min. After washing with PBS several times, the sections were incubated with 6 M urea and 100 mM glycine, pH 3.3 for 35 min, then washed 4 times with PBS. Next, the samples were blocked with 5% BSA in PBS for 1 h at room temperature. The HA-tag (C29F4) rabbit monoclonal antibody was added to the blocking solution in 1:200 dilution, and the samples were incubated overnight at 4 °C in a humidified glass chamber. On the next day, the sections were washed with PBS for 30 min then incubated with the secondary antibody (Alexa Fluor488 donkey anti-rabbit IgG (H + L) were purchased from Life Technologies, Waltham, MA, USA) at RT for 1 h in darkness. After this step, the samples were washed again with PBS for 30 min. We used Mowiol 4-88 (Sigma Aldrich, St. Louis, MO, USA) antifade reagent for mounting.

Immunostained samples were analyzed with an LSM710 confocal laser-scanning microscope (Carl Zeiss Meditec AG, Jena, Germany) with 20× and 40× objective.

### 2.7. Western Blotting and Detection of NC1 Crosslinking

To study the in vivo, proprotein convertase-mediated processing of PXDN and its NC1-crosslinking activity, protein samples were prepared from lung and kidney of wild type and HA-tagged PXDN transgenic mouse, and PXDN knockout mouse. For the detection of the collagen IV NC1 monomers and dimers, the organs were lysed in hypotonic buffer (10 mM CaCl_2_, 50 mM Hepes, pH = 7.4) containing 1mM benzamidine hydrochloride, 25 mM 6-aminocaproic acid, 1 mM phenylmethylsulfonyl fluoride. One-half of this lysate was treated with type I collagenase at 0.5 mg/mL concentration, and the digestion was performed at 37 °C. The digested samples were analyzed with Western blot, the primary antibody was Anti-Human α1(IV) NC1 clone H11 (Chondrex, Woodinville, WA, USA). The other half of the lysate was mixed and incubated for 30 min on ice with RIPA buffer supplemented with HALT Protease and Phosphatase Inhibitor Cocktail (Thermo Scientific) centrifugated at 16,100× *g* for 10 min at 4 °C. The clear supernatant was mixed with Laemmli sample buffer and boiled for 5 min at 95 °C. These lysates were used to analyze the in vivo proprotein convertase processing of the native- and the HA-tagged PXDN in the lung and kidney samples by Western blot. For protein sample preparation, the cultured cells were harvested from a 6-well plate two days after their transfection in a hypotonic buffer (10 mM CaCl_2_, 50 mM Hepes, pH = 7.4) containing 1 mM benzamidine hydrochloride, 25 mM 6-aminocaproic acid, 1 mM phenylmethylsulfonyl fluoride. One-half of this lysate was treated with type I collagenase in 0.5 mg/mL concentration, and the digestion was going on ON at 37 °C. The digested samples were analyzed with Western blot, and the primary antibody was Anti-Human α1(IV) NC1 clone H11 (Chondrex). The other half of the lysate was mixed with RIPA buffer containing HALT Protease and Phosphatase Inhibitor Cocktail (Thermo Scientific) and incubated on ice for 10 min then centrifugated at 16,100× *g* for 10 min at 4 °C. The clear supernatant was mixed with Laemmli sample buffer and boiled for 5 min at 95 °C. These lysates were used to confirm the presence of the FLAG- and V5-tagged PXDN and PXDNL in the cell lysates. The proprotein convertase inhibitor-treated and siRNA-treated cells were harvested in RIPA buffer containing HALT Protease and Phosphatase Inhibitor Cocktail (Thermo Scientific), 1 mM benzamidine hydrochloride, 25 mM 6-aminocaproic acid, 1 mM phenylmethylsulfonyl fluoride, and incubated on ice for 10 min then centrifugated at 16,100× *g* for 10 min at 4 °C. The clear supernatant was mixed with Laemmli sample buffer and boiled for 5 min at 95 °C. The protein samples derived from cell culture experiments or the mouse organs were run on 8–10% SDS polyacrylamide gels, the separated proteins were blotted on nitrocellulose membranes. The membranes were incubated for 1 h in 5% milk powder, 0.1% Tween-PBS and the first antibody was added for 1 h in the blocking buffer. After washing for 30 min with PBS containing 0.1% Tween, the membranes were incubated with the secondary antibody for 1 h (horseradish peroxidase (HRP)-conjugated anti-mouse, anti-rat, or anti-rabbit IgG). This was followed by washing the membrane again with PBS containing 0.1% Tween for 30 min. In the end, the target proteins were detected based on enhanced chemiluminescence with a Westernbright ECL kit (Advansta, San Jose, CA, USA).

## 3. Results

**Figure 1:**
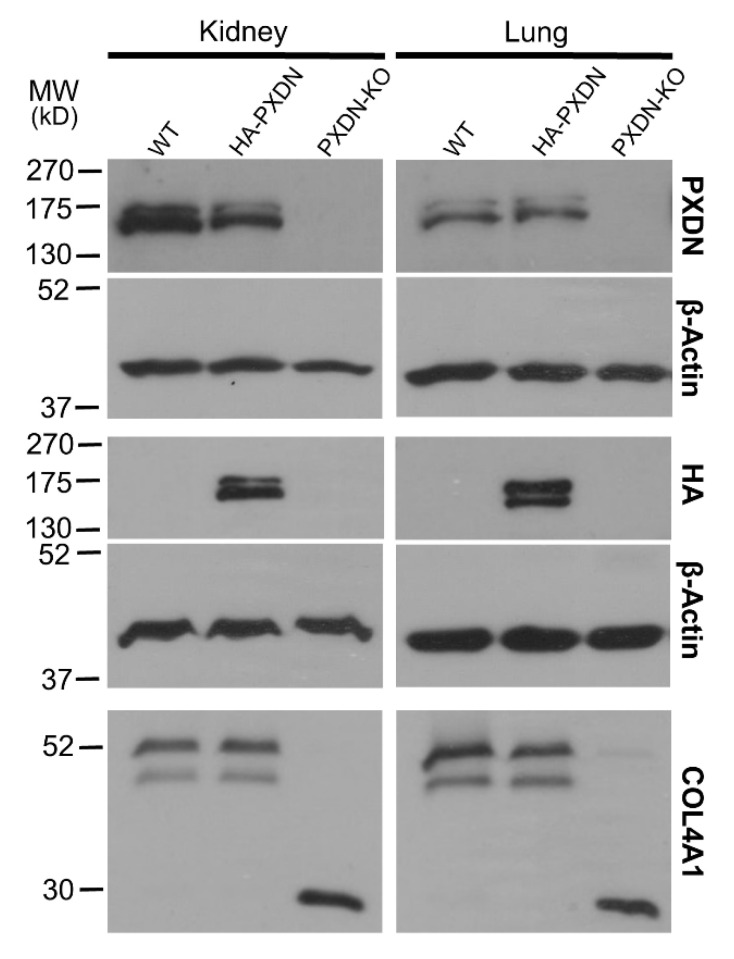
Generation of a hemagglutinin epitope (HA)-tagged PXDN expressing, knock-in mouse model. Western blot analysis of kidney-, and lung lysates prepared from WT, HA-PXDN knock-in, and PXDN-KO animals. Most upper blots show the presence of the uncleaved and cleaved PXDN with a polyclonal PXDN-specific antibody. The HA signals appear at the expected molecular weight in the knock-in samples; the upper band represents the uncleaved, the lower band indicates the proteolytically cleaved form of PXDN. The lowest blot is the analysis of collagen IV crosslinking in WT, HA-PXDN, and PXDN-KO animals. The collagenase-digested samples were separated with gel electrophoresis, and the membranes were tested for the crosslinked dimeric and uncrosslinked monomeric NC1 domains of α1 isoform of collagen IV. No difference was detected between the wild type and HA-PXDN knock-in mouse in crosslinking activity.

To facilitate the investigation of PXDN’s localization and function in mouse tissues and cells, we have developed a knock-in (KI) animal model, where we introduced a hemagglutinin (HA) epitope tag-encoding nucleotide sequence into the mouse PXDN gene, using the CRISPR/Cas9 technique [15]. We modified the PXDN gene to contain the hemagglutinin-tag at the N-terminus of the encoded protein, following the secretory signal. We hypothesized that if proteolysis of PXDN’s C-terminus also occurs in vivo, then we would detect two HA-tagged protein products with different molecular weights. As shown in Figure 1, we observed two bands with slightly different molecular weights in the kidney and lung. Double PXDN signals, with a slight difference in molecular weights, were also detected by using a polyclonal PXDN-specific antibody (Figure 1).

**Figure 2:**
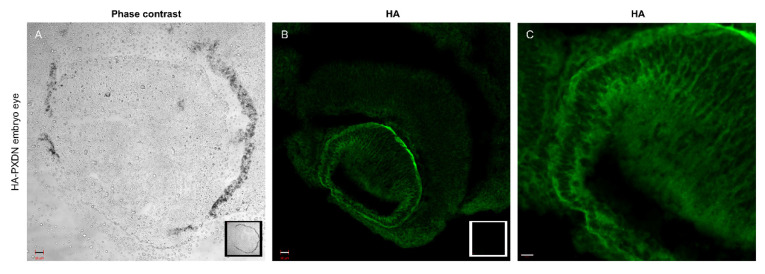
Immunostaining of PXDN in mouse embryo eye. (**A**) Phase contrast picture was taken with a 20× objective of an embryo eye prepared from HA-PXDN knock-in mouse. In the right corner of the picture, the inserted photo is made of a WT embryo eye. Bar indicates 20 µm. (**B**) Immunofluorescent staining of HA-PXDN with HA-specific antibody shows specific labeling in the developing lens, around the cells, and in basement membrane-like structure. In the right corner of the picture, the inserted photo is made of a WT embryo eye, where there is no specific signal. Bar indicates 20 µm. (**C**) Higher magnification with 40× objective of the immunostained HA-PXDN embryo eye, even more highlighting the details of the HA signal’s localization in the developing lens. Bar indicates 10 µm.

These observations suggest that proteolytic procession of PXDN also occurs under in vivo conditions. The in vivo labeling of PXDN did not alter its physiological activity, as we did not observe any phenotypic abnormalities in mice which were hetero- or homozygous for the KI allele. Additionally, collagen IV crosslinking was identical in WT and KI animals, proving that the enzymatic activity of PXDN was not affected by the modification (Figure 1, lower panel). On the other hand, we did not detect crosslinked NC1 domains in PXDN knockout tissues. With the help of the novel KI model, we could immunostain HA-PXDN in the developing mouse eye. We chose this organ for immunostaining, because PXDN is highly expressed in the developing mouse eye and essential for normal eye development in both humans and mice [17,18]. As shown in Figure 2, we detected intense PXDN staining in the eye, where the protein showed a network-like localization with strong labeling of the borders of the developing lens. No staining was observed in WT developing eyes (Figure 2).

**Figure 3:**
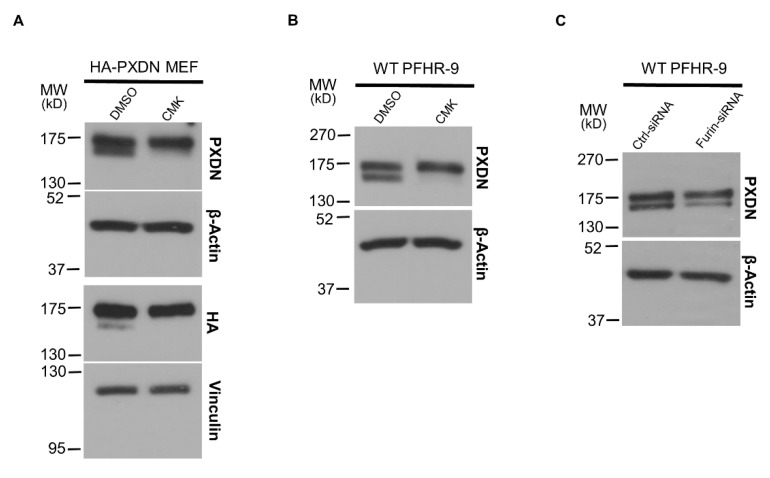
The effect of proprotein-convertase inhibitor and furin-specific siRNA treatment in cell culture. (**A**) Mouse embryonic fibroblasts (MEFs) prepared from HA-PXDN embryo were treated with furin inhibitor CMK or with the inhibitor’s solvent DMSO. The CMK treatment significantly reduced the intensity of the lower band, which represents the cleaved form of PXDN. (**B**) Treatment of WT PFHR-9 with CMK shows the same result, the inhibitor reduced the proteolytic processing of PXDN. (**C**) Treatment of WT PFHR-9 with furin-specific siRNA confirmed the role of furin in the proteolytic cleavage of PXDN.

The presence of PXDN protein signals with slightly different molecular weights in mouse tissues suggested, but did not prove the proteolytic processing of PXDN. We, therefore, prepared fibroblasts from KI embryos (mouse embryonic fibroblasts, MEFs) and studied the effect of the proprotein-convertase inhibitor Decanoyl-Arg-Val-Lys-Arg-Chloromethylketone (CMK) in cell culture. As shown in Figure 3A, we could observe the double PXDN signal in the KI fibroblasts, and the intensity of the lower band was significantly decreased after treating the cells with the furin inhibitor CMK. These observations suggested that the PXDN signal with lower molecular weight indeed represents the proteolitically-processed form of the enzyme.

After confirming the proteolysis of PXDN in tissues and primary cells, we went on to study the process in PFHR-9 cells, a mammalian cell line characterized by an intense synthesis of extracellular matrix [19]. As a critical step in ECM synthesis, PFHR-9 cells produce and crosslink collagen IV, thus offering an attractive cell model to study collagen IV turnover. Similar to that observed in mouse tissues and MEFs, we could also detect two PXDN bands with slightly different molecular weights in PFHR-9 cells, and the treatment of PFHR-9 cells with CMK inhibited PXDN processing (Figure 3B). The previous experiments did not inform us about the molecular identity of the enzyme involved in PXDN processing. We, therefore, used siRNA to test whether the proprotein-convertase furin is responsible for processing PXDN. Figure 3C shows that a furin-specific siRNA inhibited the proteolysis of PXDN, thus proving a role for furin in the process.

**Figure 4:**
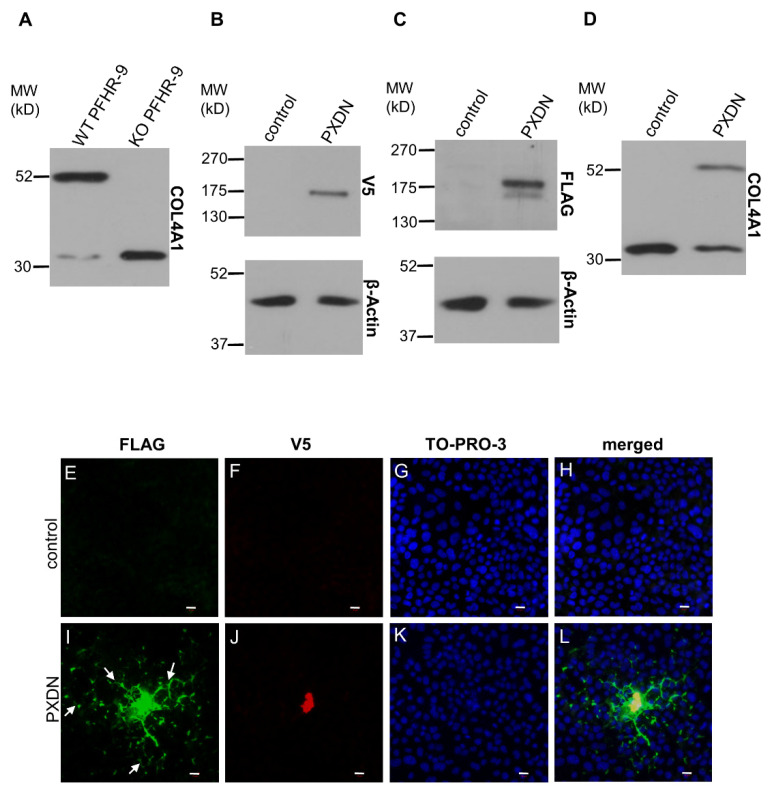
Characterization of a double-tagged PXDN in PXDN-deficient PFHR-9 cells. (**A**) Analysis of collagen IV crosslinking in WT and KO PFHR-9 cells. The collagenase-digested samples were tested for the crosslinked dimeric and uncrosslinked monomeric NC1 domains of collagen IV α1 isoform. The KO cell line lacks collagen IV crosslinking activity. (**B**) V5 signal is present at the expected molecular weight of PXDN in transfected cells. (**C**) A double FLAG signal appears in transfected cells at the molecular weight of PXDN. The upper band is uncleaved-, the lower one is the cleaved form of the protein. (**D**) Transfection of PXDN-deficient cells with the double-tagged PXDN construct rescues collagen IV crosslinking activity. (**E**–**H**) Immunofluorescent staining of PXDN-deficient, non-transfected cells (control) shows the lack of FLAG- (**E**) and V5 signal (**F**). TO-PRO-3 (blue) staining (**G**) and the merged picture (**H**) with the nuclear signal proves the presence of the cells. (**I**–**L**) PXDN-deficient cells transfected with the double-tagged PXDN construct show cell-associated and also network-like extracellular localization (arrows) of the *N*-terminally FLAG (green) tagged PXDN (**I**). The C-terminal V5 tag signal (red) is cell-associated (**J**). (**K**) TO-PRO-3 signals indicate the presence of nuclei, and on the merged picture, we can observe the partial colocalization of the FLAG- and V5 signals (**L**). The bar indicates 10 µm.

Using the CRISPR/Cas9 system, we previously created a PXDN-deficient PFHR-9 cell line [16]. PXDN-deficient cells fail to form crosslinks between NC1 domains, as demonstrated by the absence of NC1 dimers in the collagenase lysates of PXDN-deficient cells, detected by an NC1-specific antibody (Figure 4A). The PXDN KO cell line offered a promising tool to gain further information about the proteolytic processing of PXDN. We designed a PXDN construct where the encoded protein was labeled by a FLAG-tag at the N-terminus (following the signal peptide), and the protein was also marked by a C-terminal V5-epitope. Designs of all PXDN constructs used in this paper are shown in Figure S1. We transfected this construct into PXDN-deficient PFHR-9 cells and detected PXDN expression with FLAG- and V5-specific antibodies, respectively. As shown in Figure 4B, the Western blot signal obtained with the anti-V5 antibody was a single band, while two bands were recognized by the anti-FLAG antibody (Figure 4C). This experiment confirmed that the proteolytic processing of the epitope-tagged PXDN also occurs in PFHR-9 cells. The heterologous expression of PXDN in KO cells restored collagen IV crosslinking (Figure 4D), proving that the double epitope-labeled PXDN is functional in PFHR-9 cells. Using this construct offered a potentially valuable experimental tool to study the post-translational processing and function of PXDN.

**Figure 5:**
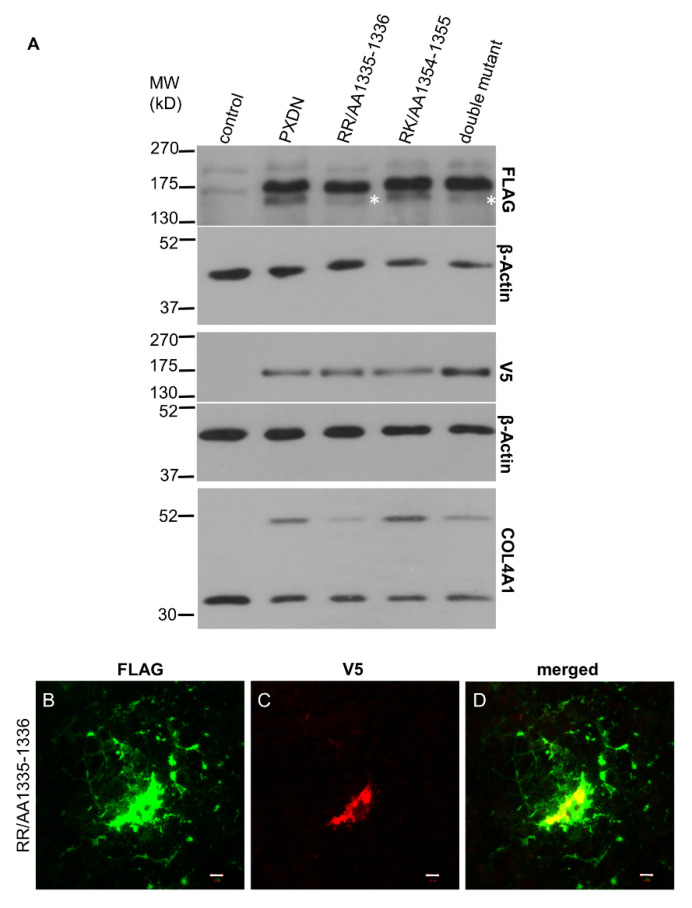
Investigation of the target sites for PXDN’s proteolysis. (**A**) Western blot analysis of PXDN-deficient cells transfected with the double-tagged WT PXDN or with different mutated PXDN constructs. In cells transfected with the RR/AA1335-1336 and the double mutant PXDN constructs, we can observe the decreased intensity of the lower band (marked by asterisks). On the lowest panel, we can observe that the RR/AA1335-1336 mutation inhibits the crosslinking activity of PXDN. (**B**–**D**) Immunofluorescent staining of RR/AA1335-1336 PXDN-transfected cells shows cell-associated and extracellular FLAG signal (green) (**B**). The V5 signal (red) appears intracellularly (**C**). The merged picture (**D**) demonstrates that the cell-associated FLAG- and V5 signals are partially colocalized. The bar indicates 10 µm.

In previous experiments, where we analyzed the localization of C-terminally labeled PXDN, we consistently observed cell-associated localization of PXDN [10]. However, the results of the experiments described above indicated that we detect only the non-processed form of PXDN, when a C-terminal tag is used for detection. Therefore, we introduced the double-tagged form of PXDN into KO PFHR-9 cells and studied the localization of the protein by confocal microscopy. In these experiments, we could detect the C-terminal V5 signal only intracellularly (Figure 4J), while the FLAG signal was observed both intra- and extracellularly (Figure 4I). Interestingly, we also detected the secreted protein relatively far from the transfected cells. Next, we aimed to examine the effect of proteolysis on the function and localization of PXDN. Using the ProP 1.0 proprotein cleavage prediction software (http://www.cbs.dtu.dk/services/ProP/), we identified two potential sites for proteolysis. One candidate site, Arg1336 was the same as the one identified by Colon et al. [14]. A distinct position, Lys1355, was also recognized by the software. We introduced mutations at these sites so that Arg1335 and Arg1336 were mutated to alanines in one construct (RR/AA 1335-1336), and Arg1354 and Lys1355 were changed to alanines in a different plasmid (RK/AA 1354-1355). We also made a construct carrying mutations at both sites. As shown in Figure 5A, the RR/AA 1335-1336 mutation reduced the proteolytic processing of PXDN, indicated by the decreased intensity of the lower molecular weight signal. On the other hand, the RK/AA 1354-1355 mutation has no detectable effect on proteolysis, and it did not modify the impact of the first mutation (Figure 5A).

To study the functional significance of the proteolysis on the enzymatic function of PXDN, we analyzed collagen IV crosslinking of cells expressing the mutant proteins (Figure 5A, lowest panel). We observed the reduced formation of NC1-dimers in cells expressing the RR/AA 1335-1336 mutant form, while the other amino acid change had no impact on crosslinking (Figure 5A, lowest panel). In subsequent experiments, we compared the localization of wild-type and mutant constructs, and we found that mutants were also deposited into the extracellular space (Figure 5B). However, the lack of the V5 signal indicated that only the processed PXDN form was deposited around the cells.

**Figure 6:**
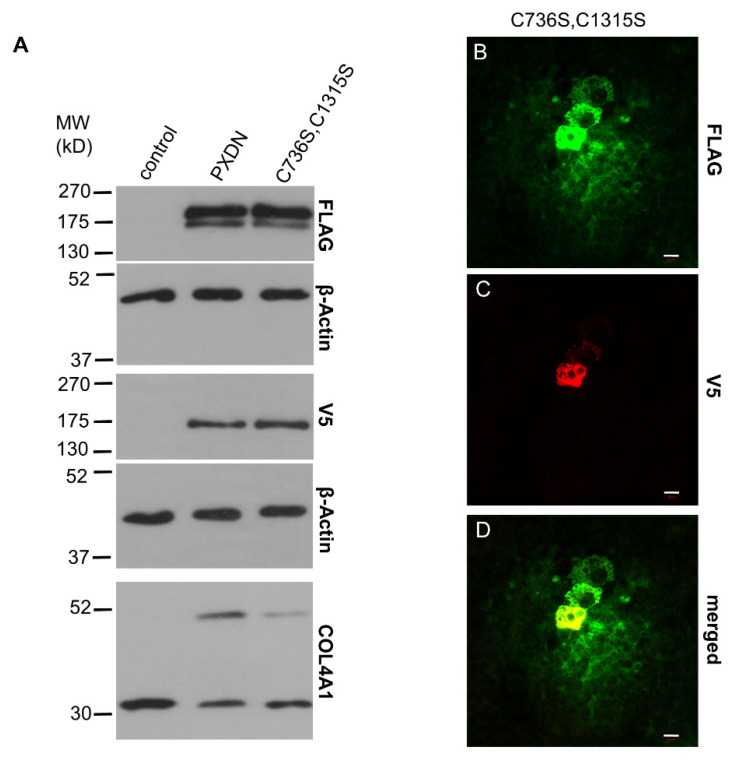
Analysis of the impact of quaternary structure formation on the proteolytic processing of PXDN. (**A**) Western blot analysis of the FLAG- and V5 signals of the trimerization mutant PXDN construct (C736S, C1315S) shows slightly reduced proteolytic processing of the protein. The lowest blot shows reduced collagen IV-crosslinking activity of the trimerization mutant PXDN. (**B**–**D**) Immunostaining of the C736S, C1315S PXDN mutant-transfected cells indicates cell-associated and diffuse extracellular FLAG signal (green) (**B**). The V5 signal (red) appears only in a cell-associated manner (**C**). The merged picture (**D**) shows the partial colocalization of the two signals. The bar indicates 10 µm.

Next, we sought to determine if the quaternary structure of PXDN is a prerequisite for the proteolytic maturation step. In our previous work, we identified two cysteines (Cys736 and Cys1315), which mediate the oligomerization of PXDN into disulfide-coupled trimers [10]. When we transfected KO PFHR-9 cells with the monomeric form of PXDN, carrying the Cys736Ser and Cys1315Ser mutations, we still observed slightly reduced proteolysis of the protein (Figure 6A). In accordance with our previous results obtained in a different cell model (10), the monomeric PXDN showed reduced crosslinking (Figure 6A, lowest panel). This mutant’s staining pattern was different from the one observed for the trimeric form, as monomeric PXDN was more dispersed around transfected cells (Figure 6B).

**Figure 7:**
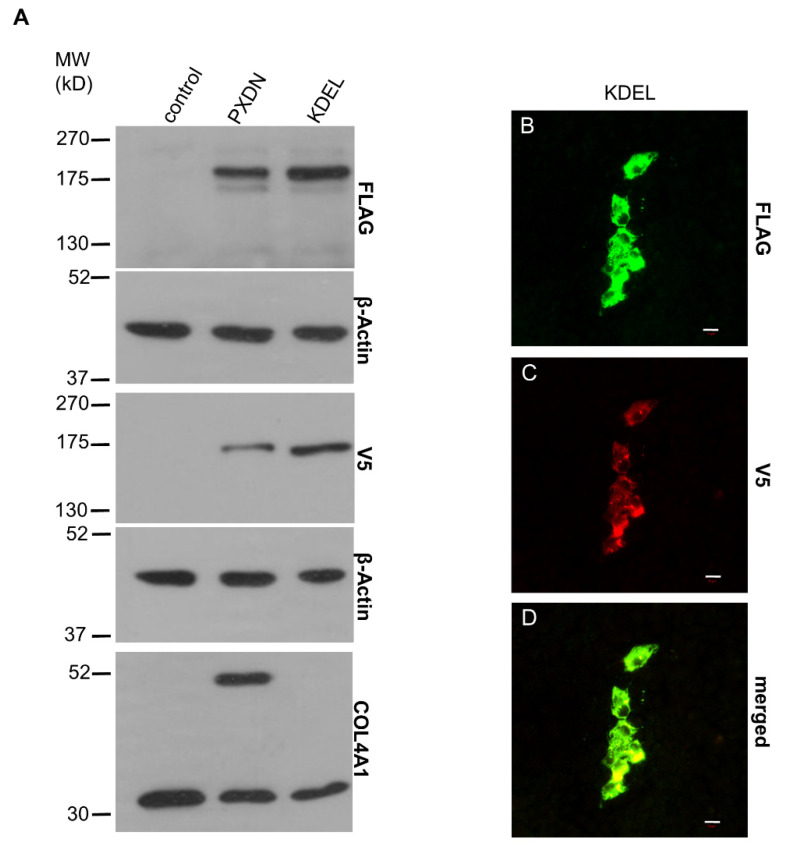
Characterization of the ER retention mutant PXDN. (**A**) Western blot analysis of the FLAG- and V5 signals of the ER retention mutant PXDN construct (KDEL) indicates reduced proteolytic processing of the protein. On the lowest blot, we can observe that the mutant protein’s collagen IV-crosslinking activity is essentially absent. (**B**–**D**) Immunostaining of the ER retention mutant PXDN transfected cells shows only cell-associated FLAG signal (green) (**B**). The V5 signal (red) also appears only in a cell-associated manner (**C**). The merged picture (**D**) shows the partial colocalization of the FLAG- and V5 signals. The bar indicates 10 µm.

The proprotein convertase-mediated cleavage of proteins can occur at different intracellular locations, including the trans-Golgi network and at other stations of the secretory pathway [20]. Association of protein conversion to the extracellular surface of the plasma membrane was also described. To assess the location of PXDN cleavage, we attached an ER retention signal (KDEL) to the C-terminus of PXDN. When this construct was expressed in PXDN-deficient PFHR-9 cells, we consistently observed a higher amount of cell-associated PXDN (Figure 7A), which is explained by the decreased secretion of the protein. The presence of the ER-retention signal decreased the proteolytic processing of PXDN and erased the crosslinking activity of the enzyme (Figure 7A, lowest panel). The absence of extracellularly localized PXDN was also confirmed in immunostaining experiments (Figure 7B,C).

**Figure 8:**
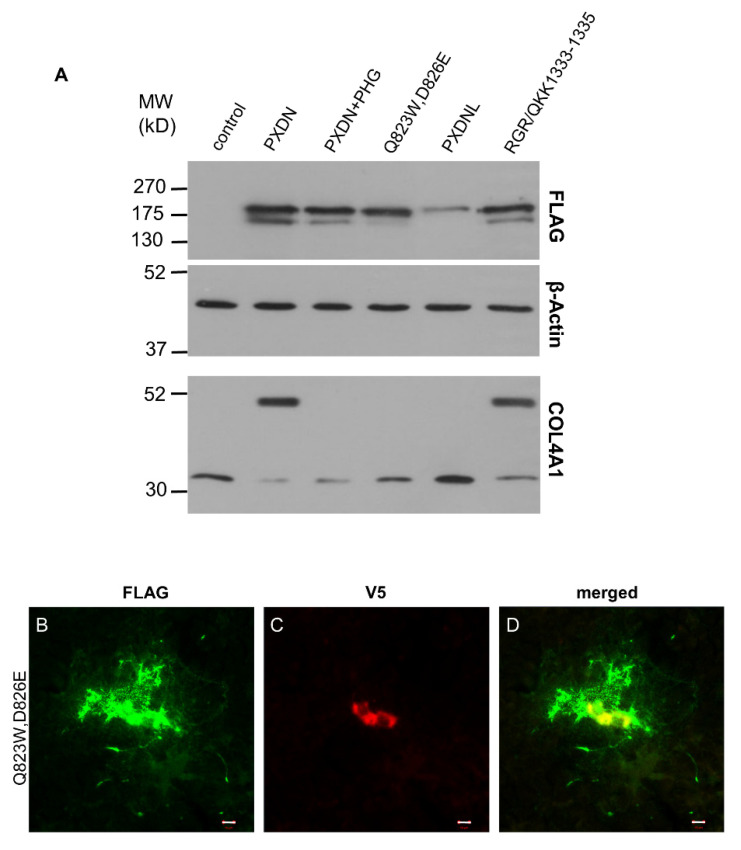
Examination of the effect of peroxidase activity on the proteolytic processing of PXDN. (**A**) Western blot analysis of cells transfected with WT PXDN (grown in the absence or presence of 50 µM PHG), peroxidase activity mutant PXDN (Q823W, D826E), PXDNL, or with PXDN mutant carrying PXDNL’s putative proprotein convertase cleavage site (RGR/QKK1333-1335). Pharmacological inhibition of enzymatic activity (PXDN+PHG) or the lack of peroxidase activity (Q823W, D826E) decreased the proteolytic processing of PXDN. PXDNL, which does not have peroxidase activity, is not processed at all. A PXDN mutant carrying the recognition site imported from PXDNL is partially processed (RGR/QKK1333-1335). On the lowest blot of this panel, we can see that PHG treatment inhibits the crosslinking activity. The peroxidase activity mutant PXDN cannot crosslink collagen IV, PXDNL doesn’t have crosslinking activity, and the RGR/QKK1333-1335 mutant PXDN has normal crosslinking activity. (**B**–**D**) Immunostaining of the peroxidase activity mutant PXDN transfected cells shows cell-associated and diffuse extracellular FLAG signal (green) (**B**). The V5 signal (red) is cell-associated (**C**). The merged picture (**D**) shows the partial colocalization of the FLAG- and V5 signals. The bar indicates 10 µm.

Next, we examined if the peroxidase activity of PXDN has any effect on its post-translational processing. The relevance of this question is augmented by the fact that PXDNL, a close homolog of PXDN, showed no measurable peroxidase activity, when it was expressed in COS-7 cells [13]. The lack of peroxidase activity is probably caused by changes in the peroxidase homology domain of PXDNL. In PXDNL, Trp808 and Glu811 replace amino acids, which are involved in halide binding (Gln823) and form a covalent link with heme (Asp826) in PXDN [9,13,21]. To study the importance of enzymatic activity in the processing of PXDN, we first exposed PXDN-transfected cells to phloroglucinol (PHG), a potent inhibitor of peroxidases. This compound was previously shown to inhibit collagen IV crosslinking in PFHR-9 cells [7]. As shown in Figure 8A, PHG reduced the proteolytic processing of PXDN. PHG treatment also inhibited the crosslinking of collagen IV (Figure 8A, lowest panel). In subsequent experiments, we transfected PXDN-deficient PFHR-9 cells with a catalytically inactive form of PXDN, in which two highly conserved amino acids in the active site were mutated (Gln823Trp and Asp826Glu). We previously demonstrated that these amino acid changes erased the peroxidase activity of PXDN [10]. As shown in Figure 8A, the absence of peroxidase activity reduced the proteolytic processing of the enzyme. As it was expected, the catalytically inactive form failed to crosslink collagen IV (Figure 8A, lowest panel). In further experiments, we studied the localization of the mutant enzyme and we found the protein to be localized similar to its WT counterpart, although we consistently observed smaller areas of FLAG positivity around transfected cells (Figure 8B).

PXDNL is a close homolog of PXDN with 58% identity and 72% similarity at the protein level [13]. Because of its high homology to PXDN, we wanted to investigate whether PXDNL is also subject to proteolysis in PFHR-9 cells. Using the proprotein cleavage site prediction software, ProP 1.0, we could identify a candidate site for proteolysis (Arg1319) at the C-terminal part of the protein of PXDNL. Although the sequence motif is different from the one in PXDN, the predicted size of the C-terminal residual product (144 amino acids) is only one amino acid longer than the corresponding fragment of PXDN (143 amino acids). We also labeled PXDNL with an *N*-terminal FLAG-tag, which helped us to track the impact of a possible cleavage on its molecular weight. Interestingly, despite the presence of a putative cleavage site, when PXDNL was expressed in KO PFHR-9 cells, we did not detect its proteolysis (Figure 8A). One possible explanation for the absence of proteolysis is that PXDNL’s cleavage site is not recognized by the proprotein-convertase of PFHR-9 cells. To test this possibility, we introduced the predicted cleavage site of PXDNL into PXDN (RGR/QKK1333-1335). As shown in Figure 8A, PXDN was efficiently cleaved, indicating that the proprotein convertase indeed recognizes the sequence motif imported from PXDNL. Results of the previously described experiments suggested that the enzymatic activity of PXDN facilitates its post-translational processing.

## 4. Discussion

Basement membranes have an essential role in providing mechanical support for cells, but beyond the structural function, they are also crucial in conveying extracellular signals [22]. The synthesis and reorganization of basal membranes are essential for normal growth and development [22,23]. Tissue regeneration, neovascularization is also characterized by basal membrane formation and remodeling. Several different components of basement membranes were identified over the years, and the discovery of PXDN has substantially increased our understanding of the biochemical processes which mediate the formation of these sheet-like ECM structures. In the reaction catalyzed by PXDN, bromide is oxidized into hypobromous acid, which mediates the oxidative crosslinking of the NC1 domains of collagen IV protomers [7,8]. This reaction contributes to the formation of a collagen IV meshwork, the core component of basement membranes. Our knowledge of PXDN function is rapidly growing, but we still know much less about this enzyme than we do about other members of the peroxidase family. In this work, we report several novel insights about the processing of PXDN and its role in ECM synthesis.

First, by developing a new animal model, we could prove that the proteolytic cleavage of PXDN occurs in vivo in all organs we have examined so far. Although we detect the proteolysis with the help of an *N*-terminal knock-in (hemagglutinin) tag, it is unlikely that this modification affected the enzymatic function of PXDN. First, the animals did not show the characteristic phenotype of PXDN-deficient mice [10,17,24]. Furthermore, we found no disturbance of collagen IV crosslinking in the KI animals, confirming that the attachment of an HA-tag to the *N*-terminal part of the protein did not interfere with the enzymatic function of PXDN. Introducing the HA-tag into PXDN also made possible the immunostaining of PXDN in the developing mouse eye. Mutations in the human and mouse PXDN genes both lead to anterior segment dysgenesis [17,18]. The severe ocular developmental defect observed in the absence of PXDN limits the use of PXDN KO animals as appropriate negative controls in immunostaining experiments. The HA-PXDN animals, on the other hand, have normal eye development (Figure 2), so this model offers an important tool to study the expression and localization of PXDN during the different stages of eye development.

In experiments where we studied the processing of PXDN in KI MEFs, we could show that the appearance of the lower molecular weight PXDN signal is effectively inhibited by the furin inhibitor CMK. These experiments proved that two PXDN bands detected in tissue lysates likely represent the proporotein-convertase-mediated conversion of PXDN. The involvement of the proprotein-convertase furin was also confirmed in PFHR-9 cells, where similar to the effect of pharmacological inhibition, we could also reduce PXDN processing by furin-specific siRNA.

To get a detailed insight into the processing of PXDN, we designed a mammalian cell-based experimental system, where we could study three critical aspects of PXDN function: (i) proteolytic procession, (ii) biochemical activity (i.e., collagen IV crosslinking), and (iii) localization. We could achieve these goals by designing a PXDN construct where two epitopes were attached to the N- and C-terminus of PXDN, respectively. We found the double epitope-labeled PXDN to be fully functional, as we could restore the missing collagen IV crosslinking in PXDN-deficient mouse embryonic carcinoma (PFHR-9) cells. One further advantage of using this system was that we could detect the secreted fraction of PXDN in the ECM. Notably, only the *N*-terminally tagged fraction appeared outside of the cells, and the C-terminal (V5) tag was exclusively observed intracellularly. This finding suggests that during collagen IV crosslinking, only the C-terminally cleaved protein becomes integrated into the ECM. This observation also explains the results of our previous attempts, where we could not detect PXDN in the extracellular environment using a polyclonal antibody raised against the C-terminal part of the protein (unpublished data).

By using the double-tagged PXDN we confirmed that proteolytic cleavage of the heterologously-expressed protein also occurs in PFHR-9 cells, and site-directed mutagenesis helped us to identify the site of the proteolysis. According to our results, the protein cleavage occurs at Arg1336, which is the same site reported by Colon et al. [14]. We also observed that the crosslinking activity of the mutant form is significantly reduced. Although mutation of the Arg1336 substantially reduced the proteolytic processing of PXDN, it did not erase it, which probably explains the detection of the mutant form in the ECM around transfected cells.

Interestingly, our experiments revealed an additional factor that affects the maturation of PXDN, and that is the peroxidase activity of the protein. Inhibition of the enzymatic activity—either by a peroxidase inhibitor or mutagenesis—resulted in reduced proteolytic processing. Also, PXDNL, which is highly homologous to PXDN, but lacks enzymatic activity, was not cleaved in PFHR-9 cells. These results point to the existence of a previously unrecognized protein quality control mechanism in PXDN-secreting cells, limiting the maturation of catalytically-deficient forms of the enzyme. Since bromide seems to be the physiological substrate of PXDN [8,25], the oxidation of this halide may have a role in the quality control process. Alternatively, it is also possible that oxidation of a hitherto unknown protein substrate regulates the quality control mechanism. Furthermore, we can not exclude the possibility that in the absence of peroxidase activity, a local increase of H_2_O_2_ level occurs, which might interfere with the enzymatic activity of furin [26]. In this regard, it is interesting to note that PHG- and mutation-induced inhibition of peroxidase activity were equally effective in the inhibition of collagen IV crosslinking, but the impact of the peroxidase domain mutation was more prominent on the proteolytic cleavage of PXDN (Figure 8A). Heme binding was identified to be an essential step in MPO biosynthesis, as inhibiting heme synthesis blocks the maturation of MPO [27]. This observation suggests that intact catalytic activity may also be a prerequisite for the maturation of the other animal heme peroxidases.

In this study, we also analyzed whether PXDNL, a human homolog of PXDN, undergoes proteolytic cleavage in PFHR-9 cells. In our experiments, we found that PXDNL is not cleaved in PFHR-9 cells, even though it contains a sequence motif, which is efficiently recognized by proprotein convertases. This observation provides further evidence for the importance of peroxidase activity in the proteolytic processing of PXDN and PXDNL. PXDNL is highly homologous to PXDN; however, it is enzymatically inactive due to amino acid changes at positions essential for the catalytic activity of peroxidases. PXDNL is highly expressed in the human heart, pointing to a role independent of peroxidase activity, presumably through other protein domains.

## 5. Conclusions

In this study—using novel in vitro and in vivo models—we provide direct evidence that both the secretion and proteolytic cleavage are important for the collagen IV crosslinking activity of peroxidasin (PXDN). Furthermore, we also revealed that the proteolytic processing largely depends on the peroxidase activity of PXDN. A close homolog of PXDN, peroxidasin-like protein (PXDNL), which lacks peroxidase activity, is not processed by proteolytic enzymes despite the presence of cleavage recognition site in its sequence. In summary, our observations are consistent with a model that proteolysis of the enzymatically active form of PXDN occurs intracellularly at a late stage of secretion and the truncated form catalyzes collagen IV crosslinking and integrates into the ECM.

## Data Availability

The data presented in this study are avalibale in this article.

## Supplementary Materials

The following are available online at https://www.mdpi.com/article/10.3390/antiox10101565/s1, Figure S1: Schematics of different PXDN/PXDNL constructs used in the study.

## Abbreviations

ECM: extracellular matrix, EPO: eosinophil peroxidase, LPO: lactoperoxidase, MPO: myeloperoxidase, NC1: non-collagenous, PHG: phloroglucinol, PXDN: peroxidasin, PXDNL: peroxidasin-like protein, ROS: reactive oxygen species, TPO: thyroid peroxidase.
