# Supplementary figures and images for: Characterization of the Proprotein Convertase-Mediated Processing of Peroxidasin and Peroxidasin-like Protein
Source: Antioxidants (Basel). 2021 Sep 30;10(10):1565. doi: 10.3390/antiox10101565 (PMC8533639; doi:10.3390/antiox10101565)

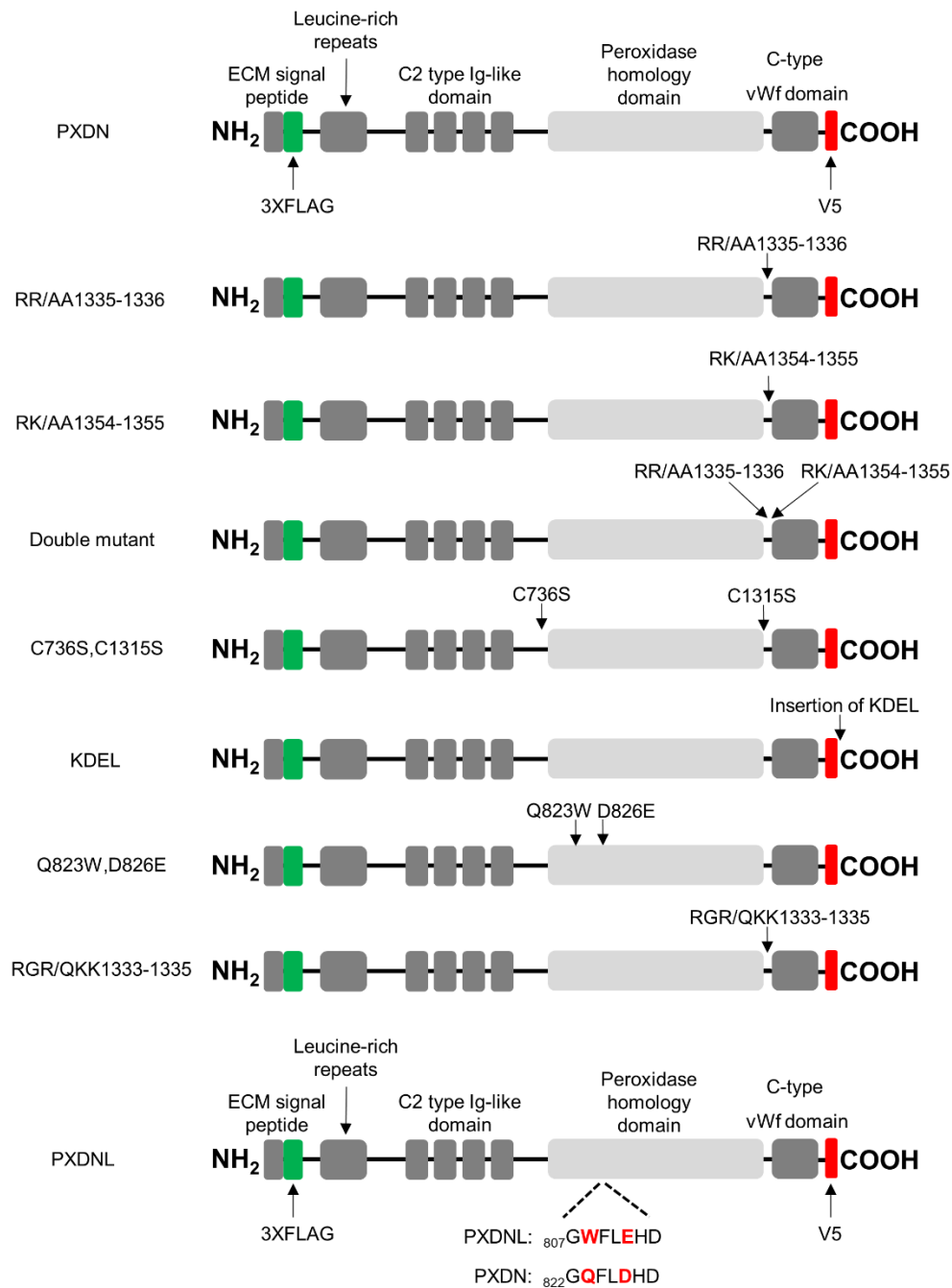

**Figure S1.** Schematics of different PXDN/PXDNL constructs used in the study.

Supplement: Supplementary file 1 [file antioxidants-10-01565-s001.zip › antioxidants-1333586-supplementary.pdf]
